# Kallmann syndrome in a patient with Weiss–Kruszka syndrome and a *de novo* deletion in 9q31.2

**DOI:** 10.1530/EJE-20-1387

**Published:** 2021-04-28

**Authors:** Anna-Pauliina Iivonen, Juho Kärkinen, Venkatram Yellapragada, Virpi Sidoroff, Henrikki Almusa, Kirsi Vaaralahti, Taneli Raivio

**Affiliations:** 1 Department of Physiology, Stem Cells and Metabolism Research Program, Faculty of Medicine, Research Programs Unit, University of Helsinki, Helsinki, Finland; 2 Pediatric Research Center, New Children’s Hospital, Helsinki University Hospital, Helsinki, Finland; 3 North Karelia Central Hospital, Joensuu, Finland; 4 Institute for Molecular Medicine Finland, FIMM, University of Helsinki, Helsinki, Finland

## Abstract

Patients with deletions on chromosome 9q31.2 may exhibit delayed puberty, craniofacial phenotype including cleft lip/palate, and olfactory bulb hypoplasia. We report a patient with congenital HH with anosmia (Kallmann syndrome, KS) and a *de novo* 2.38 Mb heterozygous deletion in 9q31.2. The deletion breakpoints (determined with whole-genome linked-read sequencing) were in the *FKTN* gene (9:108,331,353) and in a non-coding area (9:110,707,332) (hg19). The deletion encompassed six protein-coding genes (*FKTN*, *ZNF462*, *TAL2*, *TMEM38B*, *RAD23B*, and *KLF4*). *ZNF462* haploinsufficiency was consistent with the patient’s Weiss–Kruszka syndrome (craniofacial phenotype, developmental delay, and sensorineural hearing loss), but did not explain his KS. In further analyses, he did not carry rare sequence variants in 32 known KS genes in whole-exome sequencing and displayed no aberrant splicing of 15 KS genes that were expressed in peripheral blood leukocyte transcriptome. The deletion was 1.8 Mb upstream of a KS candidate gene locus (*PALM2AKAP2*) but did not suppress its expression. In conclusion, this is the first report of a patient with Weiss–Kruszka syndrome and KS. We suggest that patients carrying a microdeletion in 9q31.2 should be evaluated for the presence of KS and KS-related features.

## Introduction

Congenital hypogonadotropic hypogonadism (CHH) is a rare genetic disease that prevents pubertal development and elicits infertility due to deficient secretion or action of gonadotropin-releasing hormone (GnRH) (1). CHH is called normosmic (nCHH) if the patient has a normal sense of smell, and Kallmann syndrome (KS) if the patient has an absent or deficient ability to smell (1). Both nCHH and KS may manifest with accompanying anomalies, such as bone abnormalities, hearing impairment and ear abnormalities, cleft lip or palate, and anophthalmia and/or coloboma (1). These diseases present wide phenotypic and genetic heterogeneity, as over 60 genes underlying nCHH and KS have been identified to date (1, 2). However, several nCHH and KS genes remain to be discovered, since the currently known genes account for only half of all cases (1). nCHH and KS might be inherited in a Mendelian or oligogenic fashion depending on the causative gene; large cohort studies suggest that a significant proportion of the cases are oligogenic (1).

Recently, Weiss and Kruszka reported a series of patients with developmental delay, distinct craniofacial phenotypes, and hearing loss and showed that this syndrome is due to loss-of-function *ZNF462* mutations or microdeletions in the 9q31.2 area (3). We report a patient with a complex phenotype including Weiss–Kruszka syndrome, KS, and a *de novo* deletion on chromosome 9q31.2. The deletion encompassed *ZNF462*, *FKTN*, *TAL2*, *TMEM38B*, *RAD23B*, and* KLF4*. To date, 25 patients with Weiss–Kruszka syndrome have been reported (3, 4, 5, 6), yet delayed puberty, anosmia, or CHH are not among the listed phenotypic features. On the other hand, deletions in the 9q31.2 chromosomal area have been continuously described since the 1970s (7, 8, 9, 10, 11, 12, 13, 14, 15), at least one of whom had CHH (11), olfactory bulb hypoplasia (14), or delayed puberty in multiple family members (15), and at least four had cleft lip or palate (8, 10, 13, 14), that is, phenotypic features reminiscent of KS. However, no clear link between the 9q31.2 deletions and complete KS currently exists. The closest connection comes from disruption or pathogenic variants in the closely located *PALM2AKAP2* locus. Indeed, *PALM2* and *AKAP2*, which can form fusion transcripts, constitute potential KS disease genes, since a female with KS and Graves’ disease carried a missense mutation, which was predicted to be deleterious, in *PALM2* (16), and a male with KS and bone anomalies carried a balanced chromosomal translocation that disrupted *AKAP2* expression (17).

To investigate the putative relationship between the proband’s 9q31.2 deletion and KS, we first defined the exact deletion breakpoints with linked-read whole-genome sequencing. Next, we investigated if the deletion was associated with decreased expression of *PALM2AKAP2* and screened this gene in a set of Finnish KS patients. Since these investigations did not support the connection between *PALM2AKAP2* and KS, we performed whole-exome and RNA sequencing in the proband and his family members to exclude defects in genes implicated in KS and to reveal potential new candidate genes. Our results, together with the CHH and KS-related phenotypes in previous patients with 9q31.2 deletions suggest that microdeletions in this chromosomal region underlie KS.

## Subjects and methods

### Subjects

**Figure 1:**
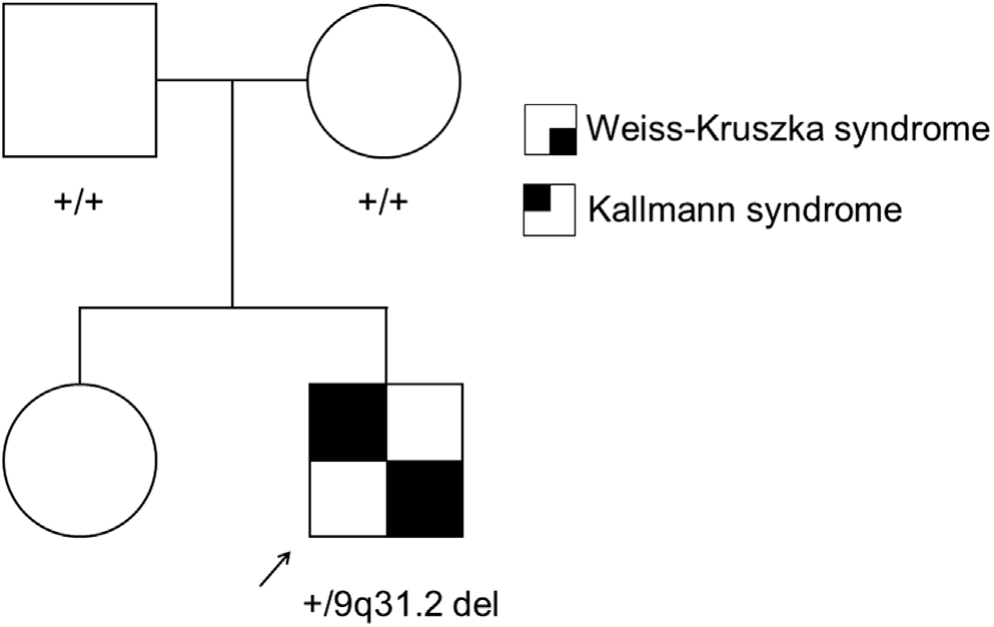
Pedigree of the family, in which the proband (*arrow*) carried a heterozygous *de novo* 2.38 Mb deletion in chromosome 9q31.2 that encompassed *FKTN*, *TAL2*, *TMEM38B*, *ZNF462*, *RAD23B*, and* KLF4*. The proband had Weiss–Kruszka syndrome due to *ZNF462* haploinsufficiency with ptosis, flat nasal tip, low set ears, mild developmental delay, and mild bilateral sensorineural hearing loss, and Kallmann syndrome (congenital hypogonadotropic hypogonadism, anosmia, and the absence of olfactory bulbs).

We investigated a Finnish family whose son (the proband) had been assessed for delayed development and subsequently for the absence of puberty. He was the second child of healthy nonconsanguineous parents (Fig. 1), and he had a healthy sister, who had normal timing of puberty since she reached menarche at the age of 12 years. The mother’s menarche age had been 11 years, and the father had no reported history of delayed puberty. Following an uneventful pregnancy, the proband was born at gestation week H42+1. His birth weight was 3240 g and his length was 50 cm. He received 4, 6, and 9 Apgar points at the ages of 1, 5, and 10 min, respectively. He was noted to have a muscular ventricular septal defect that closed spontaneously. His testes were normally descended. Before going to school, he was diagnosed with attention deficit disorder and mild developmental delay. He had distinctive facial features including ptosis, flat nasal tip, low set ears, and mild bilateral sensorineural hearing loss with normal semicircular canals in MRI. At the age of 6.5 years, he underwent comparative genetic hybridization (Agilent 44K), in which a 2.38 Mb deletion in the 9q31.2 area was detected, yet the precise breakpoints remained undetermined. The deletion was *de novo*, as his parents tested negative for the presence of the deletion.

At the age of 13.5 years, the proband was referred for the evaluation of delayed puberty and was noted to have a hypoplastic scrotum and testes. He had self-reported anosmia, which had been well appreciated in the family and absent olfactory bulbs in the MRI. He had no synkinesia, hand or foot deformities, pigmentation defects or missing teeth, nor he reported problems with balance. Abdominal ultrasound revealed two kidneys. His circulating reproductive hormone levels were low (LH 0.1 IU/L, FSH 0.2 IU/L, testosterone 0.7 nM, and inhibin B level was very low, 14 ng/L). At the age of 14 years and 11 months, he was still prepubertal (Tanner stage G1) with pubic hair stage P3. His testes were very small, only 1 cm in length (corresponding to < 1 mL). His reproductive hormone levels were still low; serum LH 0.1 IU/L, inhibin B 23 ng/L, and testosterone 0.4 nM. Based on the very small testicular size, low reproductive hormone and inhibin B levels, anosmia, and the absence of olfactory bulbs in the MRI scan, he was prepubertal, and a diagnosis of Kallmann syndrome was set (1, 18). Induction of his puberty with low-dose testosterone was commenced. During the treatment, his testis size remained extremely small (assessed at the age of 16 years and 9 months) in the setting of low gonadotropin and inhibin B (23 ng/L at the age of 15 years and 9 months; 29 ng/L at 16 years 9 months, and 28 ng/L at 17 years 7 months) levels consistent with the KS diagnosis. As a part of the diagnostic procedure, he underwent BluePrint Genetics® Kallmann Syndrome Panel Plus (version 3), a diagnostic platform that covers the coding regions of the following genes: *ANOS1*, *CHD7*, *FGF8*, *FGFR1*, *GNRHR*, *KISS1R*, *PROK2*, *PROKR2*, and *TACR3* up to20 bp of the intronic sequence with single nucleotide changes, small indels up to 220 bp, and copy number variations defined as single exon or larger deletions and duplications.

### Determination of the deletion breakpoints and presence of structural variants in KS genes with whole-genome linked-read sequencing

DNA of all family members was extracted from peripheral blood leukocytes (PBL). The proband and parent RNA were isolated with the QIAamp RNA Blood Mini Kit (QIAGEN) from PBLs. The whole-genome linked-read sequencing of the proband was performed at FIMM (Institute for Molecular Medicine Finland) according to 10× Genomics Chromium library preparation (Chromium Genome Reagent Kits v2 RevB; 10× Genomics, Pleasanton, CA, USA). The sample was sequenced with NovaSeq 6000 system (Illumina Inc., San Diego, CA, USA) using S4 flow cell and XP workflow. Read length for the paired-end run was 2×151. The reads were aligned to the GRCh37 (hg19) reference genome. The data were analyzed with Longranger 2.2.2 WGS pipeline with default parameters and GATK for variant calling and visualized with Loupe 2.1.1 (both programs by 10× Genomics). The genome was covered with a mean depth of 34.7×, and a mean molecule length of 26.8 kb. Loupe interactive visualization tool 2.1.1 was employed to define the exact 9q31.2 deletion breakpoints. In addition, as MLPA (multiplex ligation-dependent probe amplification) is available for only a fraction of known KS genes, we verified the absence of genomic structural variants of over 40 bp in size in the coding regions of 32 known KS genes (*ANOS1*, *FGFR1*, *FGF8*, *FGF17*, *PROK2*, *PROKR2*, *CHD7*, *NSMF*, *HS6ST1*, *WDR11*, *SEMA3A*, *SEMA7A*, *PLXNA1*, *SOX10*, *IL17RD*, *FEZF1*, *NDNF*, *TCF12*, *TUBB3*, *DCC*, *SMCHD1*, *KLB*, *NTN1*, *SPRY4*, *PTCH1*, *FLRT3*, *AMH*, *DUSP6*, *PLXNA3*, *NRP1*, *SPRY2*, and* NRP2*) with Loupe 2.1.1.

### Investigation of *PALM2AKAP2* expression with RT-qPCR

The RNA extracted from the proband and his parents were converted into cDNA using the iScript cDNA Synthesis Kit (Bio-Rad) according to the manufacturer’s instructions in a regular thermocycler with 1 µg of total RNA. The synthesized cDNAs were used as templates to assess the mRNA expression with a ready-to-use qPCR mix (Solis Biodyne) in a quantitative PCR machine (Roche LC480 II). The expression levels of *PALM2AKAP2* were normalized to *GAPDH*. The relative expression levels were standardized against a healthy unrelated control sample, which was given an arbitrary value of 1.0. The PCR conditions and primers are available on request.

### Sanger sequencing of *PALM2AKAP2* in Finnish Kallmann syndrome patients

We examined a set of 16 Finnish KS patients (15 men, 1 woman) without mutations in *ANOS1*, *FGFR1*, *FGF8*, *PROK2*, *PROKR2*, *CHD7*, and *WDR11* (19, 20). The exons and exon-intron boundaries of *PALM2* (NC_000009.11, NM_053016.6, GRCh37) and *AKAP2* (NC_000009.11, NM_001198656.1, GRCh37) were amplified with PCR from the genomic DNA. The PCR conditions and primers are available upon request. The PCR products were purified with ExoProStar treatment (GE Healthcare Life Sciences), and sequenced from both directions using the ABI BigDyeTerminator Cycle Sequencing Kit (v3.1) and ABI Prism 3730xl DNA Analyzer automated sequencer (Applied Biosystem). The DNA sequences were aligned and read with Sequencher® 4.9 software (Gene Codes Corporation, AnnArbor, MI, USA). As one of the patients harbored a variant NC_000009.11:g.112918586C>T, in intron 2 of *AKAP2*, his RNA was converted into cDNA by using the SuperScript® III First-Strand Synthesis System for RT-PCR kit (Invitrogen by Life Technologies) according to the manufacturer’s instructions. Part of the *AKAP2* transcript was PCR-amplified with cDNA-specific primers and subsequently sequenced. Effect of the *AKAP2* variant NC_000009.11:g.112918586C>T was also predicted with Human Splicing Finder (https://www.genomnis.com/access-hsf) (21), MutationTaster (http://www.mutationtaster.org/) (22), NetGene2 (http://www.cbs.dtu.dk/services/NetGene2/) (23, 24), and BDGP NNSPLICE (https://www.fruitfly.org/seq_tools/splice.html) (25), and its clinical interpretation was performed with InterVar (http://wintervar.wglab.org/) (26).

### Whole-exome sequencing

The whole-exome sequencing (WES) of the proband’s family was performed with Illumina Novaseq S2 PE100 technology. First, the adapter was trimmed from the reads, as well as any low-quality nucleotides from the 5’ or 3’ ends of the read, removing pairs with less than 36 bp. The reads were aligned to the GRCh37 (hg19) reference genome with the BWA (Burrows–Wheeler Aligner). Non-unique read pairs and non-unique single reads were removed and GATK Base Recalibrator was used to clean the alignment. Any potential PCR duplicates were removed using Picard MarkDuplicates, and GATK IndelRealigner was used for indel sites. The mpileup from the SAMTOOLS package was used for variant calling (27). The sequencing yielded a mean target coverage of 222× and 98% of 20× coverage.

### Data analysis

First, we verified that the coding exons of 32 KS-associated genes (*ANOS1*, *FGFR1*, *FGF8*, *FGF17*, *PROK2*, *PROKR2*, *CHD7*, *NSMF*, *HS6ST1*, *WDR11*, *SEMA3A*, *SEMA7A*, *PLXNA1*, *SOX10*, *IL17RD*, *FEZF1*, *NDNF*, *TCF12*, *TUBB3*, *DCC*, *SMCHD1*, *KLB*, *NTN1*, *SPRY4*, *PTCH1*, *FLRT3*, *AMH*, *DUSP6*, *PLXNA3*, *NRP1*, *SPRY2*, and* NRP2*) were covered in WES by using the BasePlayer analysis and visualization tool (28). Subsequently, the proband’s VCP file, provided by FIMM (27), was annotated using ANNOVAR (https://doc-openbio.readthedocs.io/projects/annovar/en/latest/). We defined that a potentially causative variant should be in the consensus splice-site or it should be nonsynonymous and not classified as benign by any of the ten applied *in silico* tools (SIFT, LRT, MutationTaster, MutationAssessor, FATHMM, PROVEAN, MetaSVM, MetaLR_pred, M-CAP, and fathmm-MKL). Additionally, a potentially causative variant(s) should (i) occur only in the proband (*de novo*) (and have a minor allele frequency MAF <0.1%); (ii) be biallelic (autosomal recessive inheritance) (MAF <2%); (iii) be monoallelic and inherited from one parent and be absent in the sister (autosomal dominant) (MAF <0.1%); or (iv) be monoallelic and inherited in the X chromosome (X-linked recessive) (MAF <2%). The frequency criteria were applied for all ethnic subpopulation frequencies in gnomAD, ExAC, 1000 Genomes, and Exome Variant Server (provided in the ANNOVAR annotation) and in dbSNP (https://www.ncbi.nlm.nih.gov/snp/) (29) (manually verified). Finally, to be accepted for further analyses, a potentially causative variant in WES had to be present in the RNA sequencing data and/or the linked-read WGS data. We confirmed the genotype and segregation of the two such variants in all family members with Sanger sequencing. The primers were designed for sequences around candidate variants in *RIMBP3C* (NC_000022.10, NM_001128633.2) and *SARS1* (NC_000001.10, NM_006513.4) (GRCh37). The PCR conditions and primers are available upon request.

### RNA sequencing

The quality and integrity of the RNA were assessed with high sensitivity D5000 Screen Tape® System (Agilent). Following the quality control, RNASeq libraries were prepared using NEBNext Ultra II Directional Poly (A) Capture Kit. RNA sequencing was carried out as paired-end sequencing with Illumina NextSeq 500 Mid Output System. RNA quality-testing, library preparation, and sequencing were performed in the Functional Genomics Unit (FUGU) at the University of Helsinki, Finland.

### RNA-sequencing data analysis

Raw adaptor-trimmed FASTQ files were subjected to quality assessment with the FASTQC tool (Simon Andrews, Babraham Bioinformatics). Based on the overall data quality and the individual reads, additional trimming was unnecessary. The sequences were aligned to the GRCh37 human genome version with the STAR package (30). Splicing events of known KS genes were visually verified with the Sashimi plot feature of the MISO framework (31) in the IGV browser from Broad Institute (32).

### Statistics and the probability estimate for the occurrence of a 9q31.2 microdeletion and KS

**Figure 2:**
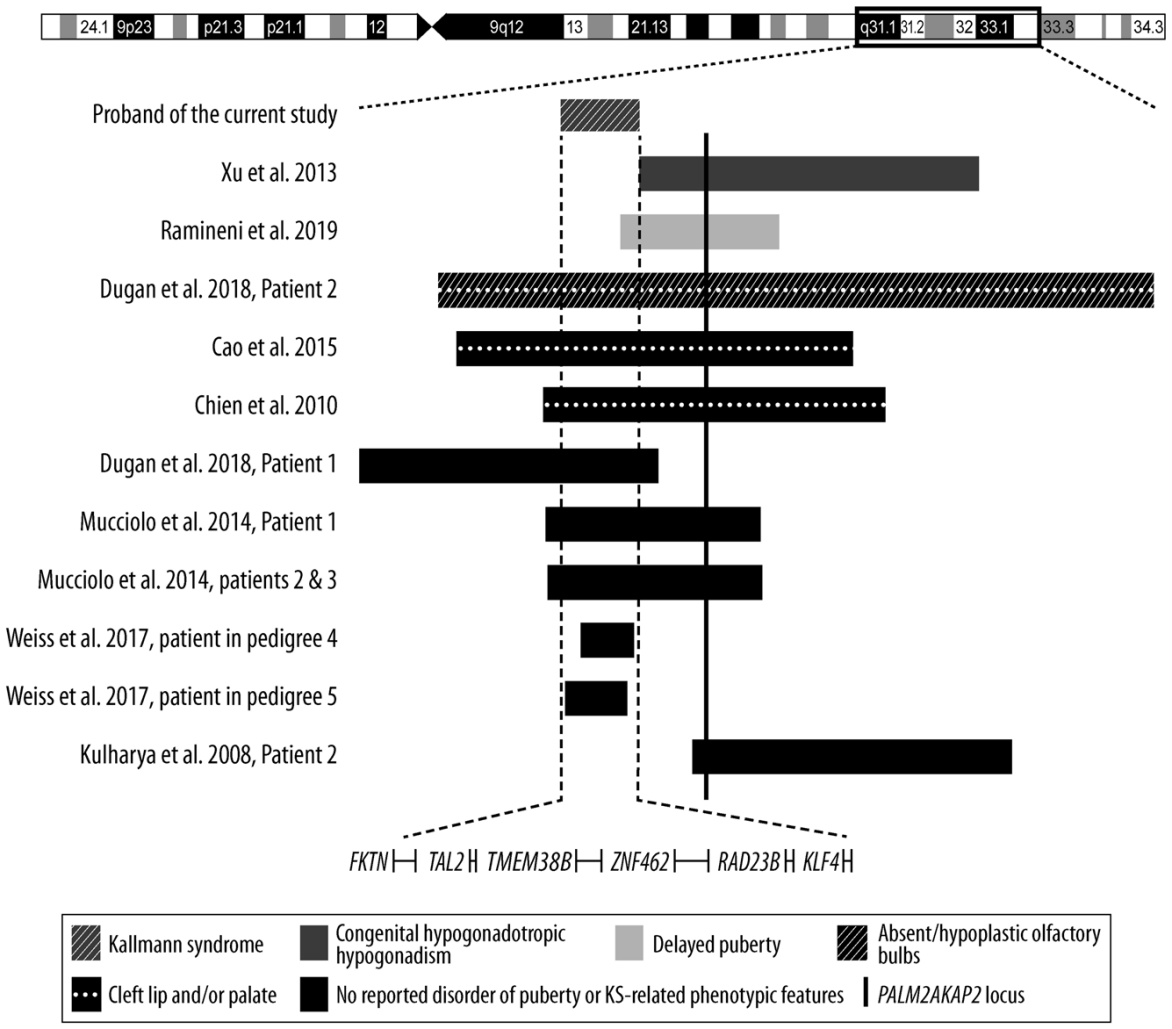
Schematic representation of the proband’s 9q31.2 deletion in relation to other informative patients with deletions encompassing this area. The dashed vertical lines indicate our proband’s 2.38 Mb deletion (chr9:108,331,353–110,707,332) (hg19), which encompassed six protein-coding genes (*FKTN*, *TAL2*, *TMEM38B*, *ZNF462*, *RAD23B*, and *KLF4*). Rectangles indicate relative locations and sizes of deletions in various patients, their colors and patterns highlight important Kallmann syndrome-related phenotypes and the vertical line indicates a Kallmann syndrome candidate genelocus *(PALM2AKAP2*). A summary of the patients is presented in Supplementary Table 2. The figure is modified from (14). The deletion coordinates are presented as GRCh37 (hg19). For Kulharya * et al.* (patient 2), Chien * et al.*, and Ramineni* et al.* patients the UCSC genome coordinate conversion tool https://genome.ucsc.edu/cgi-bin/hgLiftOver was employed to convert hg18 coordinates to hg19.

The relative expression values of *PALM2AKAP2* in the PBLs of the proband (*n* = 4) and his parents (*n* = 4) were compared with one-way ANOVA, and followed by Tukey’s HSD *post-hoc* analysis. *P*-value < 0.05 was accepted to indicate statistical significance. To evaluate the role of the 9q31.2 microdeletion in our proband, we utilized the CNV data obtained from the UK Biobank (33, 34, 35), in which 4 deletions, 1 Mb or larger in size, in a set of 472 734 people, overlapped the 24 Mb region, which is depicted with a rectangle on chromosome 9 in Fig. 2 (chr9:102,253,143–126,253,089, GRCh37). Thus, the estimated probability for our proband not to harbor a causal mutation in known KS genes and to carry such a microdeletion by chance would be very small (1:2 × 4:472 734 = 4.23e-06). Moreover, at the population level, the probability to encounter a Finnish male with KS (incidence 1:30 000) (19) without a causative mutation in KS genes, who would carry such a microdeletion would be even several magnitudes smaller (i.e. 1:30 000 × 1:2 × 4:472 734 = 1.41e-10).

## Results

We investigated the genetic and phenotypic features of a patient with KS and Weiss–Kruszka syndrome who carried a 2.38 Mb deletion on chromosome 9q31.2. A diagnostic-targeted sequencing panel for KS that covered the coding exons of genes *ANOS1*, *CHD7*, *FGF8*, *FGFR1*, *GNRHR*, *KISS1R*, *PROK2*, *PROKR2*, and *TACR3* up to 20 bp intronic sequence and structural variants was negative for likely pathogenic variants.

### Determination of the 9q31.2 deletion breakpoints

To define the exact deletion breakpoints, we performed a whole-genome linked-read sequencing of the proband’s DNA. He carried a 2.38 Mb deletion on chromosome 9:108,331,353–110,707,332, excluding *PALM2AKAP2* locus that is located at 9:112,542,589–112,934,792 (hg19) (Fig. 2). Instead, the deletion disrupted *FKTN*, which contains ten exons, at 9:108,331,353 in the first intron, and no other known gene at 9:110,707,332. The deletion encompassed six protein-coding genes (*FKTN*, *TAL2*, *TMEM38B*, *ZNF462*, *RAD23B*, and *KLF4*), none of which has been implicated in KS.

### *PALM2AKAP2* expression in peripheral blood leukocytes

**Figure 3:**
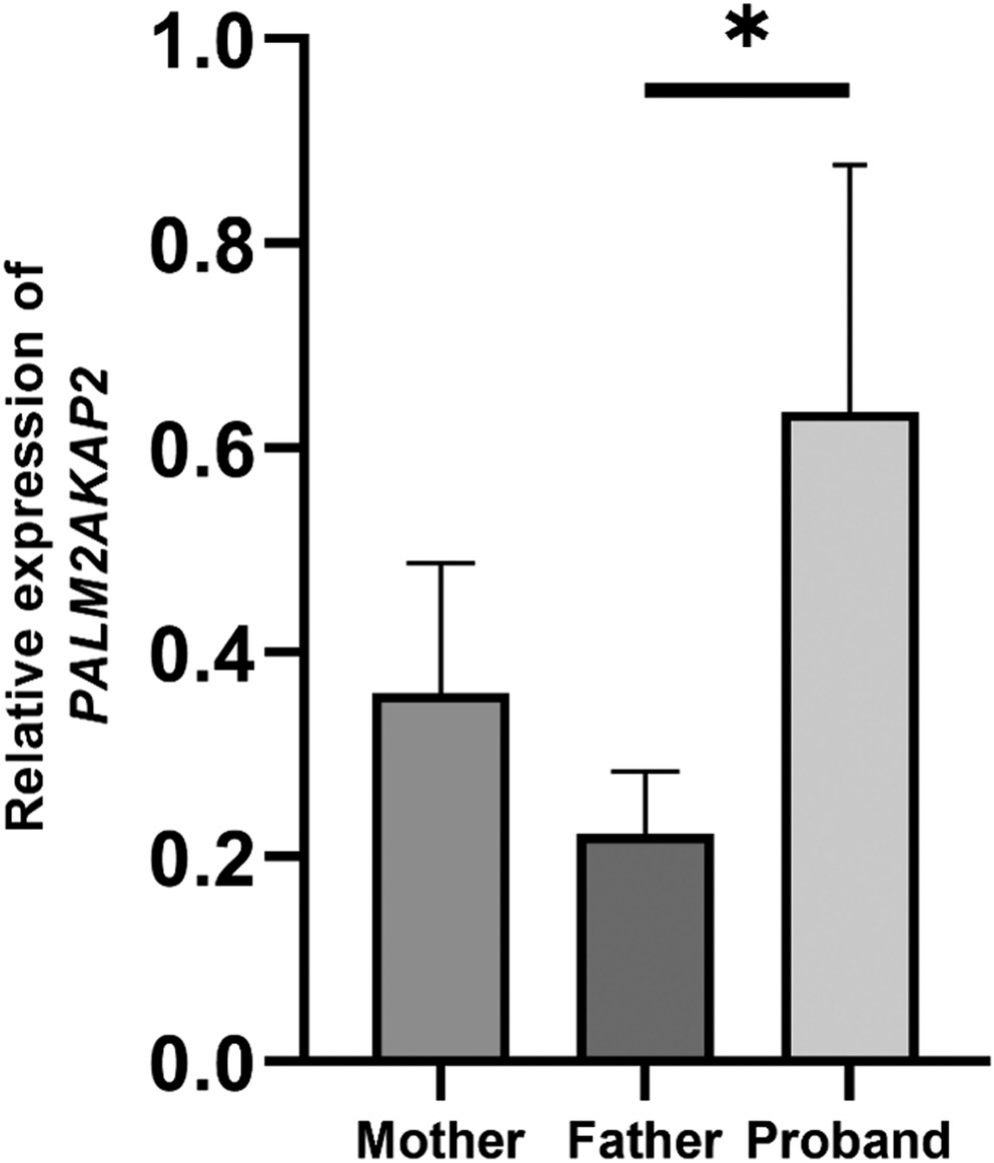
Relative expression of *PALM2AKAP2* in the peripheral blood leukocytes of the proband with Kallmann syndrome, Weiss–Kruszka syndrome, and a heterozygous *de novo* deletion in 9q31.2, and his healthy parents. The bars represent four replicates of *PALM2AKAP2* expression measured by qPCR, normalized by a housekeeping gene (*GAPDH*), and adjusted against an unrelated control sample. ^*^ *P* < .05 (ANOVA followed by Tukey HSD *post-hoc* analysis).

Given that *PALM2AKAP2* has been implicated in KS and our proband’s deletion laid approximately 1.8 Mb upstream of the *PALM2AKAP2* locus (Fig. 2), we investigated whether the expression of *PALM2AKAP2* was altered in peripheral blood leukocyte RNA. However, the proband’s *PALM2AKAP2* transcript was expressed at a higher level than in the father (Fig. 3), indicating that the deletion does not suppress *PALM2AKAP2* expression.

### Sanger sequencing of *PALM2* and *AKAP2* in 16 Finnish KS patients

To further probe the possible role of *PALM2AKAP2* in KS, we screened the coding exons and exon-intron boundaries of both *PALM2* and *AKAP2* in 16 Finnish KS patients without mutations in established KS-causing genes (19, 20). In the intron 2 of *AKAP2*, we found one heterozygous variant, NC_000009.11:g.112918586C>T (rs777796314), with a frequency of 0.00004267 in Finns (gnomAD, https://gnomad.broadinstitute.org/) (36). This variant was classified as likely benign according to the 2015 ACMG/AMP guidelines (InterVar, (http://wintervar.wglab.org/)) (26). The carrier was diagnosed with KS at the age of 14 years. He had sensorineural hearing loss in the right ear, micropenis, normal MRI, and alopecia at the age of 5. He had no family history of delayed puberty. Given that two out of four *in silico* tools predicted the variant to affect the acceptor site of exon 3 (MutationTaster: disease-causing; NetGene2: alteration of acceptor sites), we confirmed *in vitro* that the splicing was normal (*data not shown*). All other encountered *PALM2* and *AKAP2* variants had MAFs above 1% among gnomAD Finns.

### Whole-exome sequencing, RNA sequencing, splicing analysis, and identification of potentially causative variants

Given that the targeted KS gene panel, the deletion in 9q31.2 *per se*, or the adjacent candidate gene *PALM2AKAP2* provided no equivocal answer to our question of whether this deletion underlies KS in the proband, we sequenced the whole exome in the proband, his healthy parents and sister. First, no rare sequence variants were found in the 32 genes implicated in KS (*IL17RD*, *SPRY2*, *DUSP6*, *CHD7*, *FGFR1*, *SOX10*, *ANOS1*, *FGF8*, *FGF17*, *PROK2*, *PROKR2*, *NSMF*, *HS6ST1*, *WDR11*, *SEMA3A*, *SEMA7A*, *PLXNA1*, *FEZF1*, *NDNF*, *TCF12*, *TUBB3*, *DCC*, *SMCHD1*, *KLB*, *NTN1*, *SPRY4*, *PTCH1*, *FLRT3*, *AMH*, *PLXNA3*, *NRP1*, and* NRP2*). Secondly, we investigated in the linked-read WGS data whether the proband carried genomic structural variants of over 40 bp in the coding regions of these 32 KS genes. However, none of the genes were disrupted by structural variants. As WES may not detect non-coding variants that affect splicing, we investigated the splicing events of KS genes in the proband and his parents with RNA sequencing and subsequent visualization of transcripts. Overall, 15 KS genes, *FGFR1*, *PROK2*, *CHD7*, *NSMF*, *HS6ST1*, *WDR11*, *SEMA3A*, *SEMA7A*, *PLXNA1*, *TCF12*, *SMCHD1*, *PTCH1*, and *DUSP6*, were expressed in PBLs, and none of them displayed aberrant splicing events (*data not shown*). Finally, we investigated whether the WES data would contain new potential KS candidate gene mutations. Two rare variants of uncertain significance emerged, one in *RIMBP3C* and the other in *SARS1*. An overview of the variants is shown in Supplementary Table 1 and Supplementary Material (see section on supplementary materials given at the end of this article).

### Overlapping genetic region with our proband and the previously reported patient with CHH

Our proband’s deletion (9:108,331,353-110,707,332) and the deletion of the previously described patient with CHH (9:110,672,051–120,997,503) (11) overlapped in 9:110,672,051–110,707,332 (hg19). This region contained ribosomal pseudogene *RNA5SP293*. According to the UCSC Genome Browser (https://genome.ucsc.edu/), the following transcription factors were determined to have binding sites in this overlapping region: GATA3, MYC, JUND, CEBPB, TEAD4, GATA2, MAFF, USF2, USF1, BHLHE40, ZNF143, ATF3, and SIX5.

## Discussion

We report a patient with KS, developmental delay, ptosis, ventral septal defect, craniofacial phenotype, and mild bilateral sensorineural hearing loss without coding region mutations in the established KS genes, which was assessed by using a targeted sequencing panel, linked-read whole-genome sequencing, RNA sequencing, and WES. Instead, he carried a heterozygous *de novo* microdeletion in 9q31.2, which we estimated to be extremely rare (AF below 1/200 000). The deletion encompassed loci of six protein-coding genes (*FKTN*, *TAL2*, *TMEM38B*, *ZNF462*, *RAD23B*, and *KLF4*).

The deletion was 2.38 Mb in size and the 5’ breakpoint broke *FKTN* in the first intron. However, to the best of our knowledge, *FKTN* is not implicated in KS or CHH (37, 38). Haploinsufficiency of *ZNF462* is associated with Weiss–Kruszka syndrome (3), the most typical symptoms of which include developmental delay, ptosis, metopic ridge, facial asymmetry, corpus callosum dysgenesis, down-slanting palpebral fissures, arched eyebrows, epicanthal folds, short upturned nose with a bulbous tip, marked cupid bow/wide philtrum, low set ears, hypertelorism, and hearing loss (3, 5). Our proband, thus, clearly exhibited Weiss–Kruszka syndrome, and several putative links could connect *ZNF462* and the reproductive system. For instance, fibroblast growth factor 8, required for GnRH neuron ontogeny, also induces *Znf462* expression in the chick pre-placodal region, and hypothalamic expression of *ZNF462* decreases at puberty in non-human primates (39, 40). On the other hand, mutations in *ZNF462* have not been reported to cause delayed puberty or affect the sense of smell (3, 4, 5, 6), suggesting that *ZNF462* haploinsufficiency alone is not sufficient to explain the KS phenotype. In humans, the strongest GWAS signals that associated with the variation in the age at menarche were at 9q31.2 (rs2090409), the nearest genes to this SNP being *TMEM38B*, *FKTN*, *TAL2*, and *ZNF462* (41), as well as SNPs rs12684013, rs4452860, and rs7028916 (42) in the intergenic regions in 9q31.2 (menarche, age at, quantitative trait locus 3, i.e. MENAQ3 locus; MIM 612883), which were all deleted in our proband. In a recent GWAS study, *TMEM38B* was associated with puberty timing in both males and females (43). Moreover, despite the putative association of the 9q31.2 region to age at menarche and puberty timing in GWAS studies, mutations in the genes deleted in our proband have not been implicated in CHH or KS.

KS-related phenotypes in our proband and representative previously reported patients with 9q31.2 deletions are shown in Fig. 2 and Supplementary Table 2. There are two previous deletions that overlap with the one encountered in our proband and are associated with delayed puberty or CHH (11, 15). Ramineni * et al.* reported a family, in which a deletion in 9q31.2 segregated with delayed puberty. The overlapping region between their deletion and that of ours encompassed only one protein-coding gene, *KLF4*. *KLF4* is coexpressed with *SOX10*, a known KS gene, in the chick neural crest (44), and KLF4 interacts with β-catenin and inhibits Wnt signaling (45) by preventing β-catenin binding to TCF7L2 (46), a transcription factor implicated in the development of the hypothalamus and pituitary (47, 48). The deletion in our patient and in the family reported by Ramineni * et al.* also contained the SNP rs139300691, which is associated with the sense of smell (49). The second informative patient, originally classified to have delayed puberty by Xu * et al.*, had the onset of puberty at the age of 18 years, and his Tanner stage was G2 at the age of 20 in the setting of normal gonadotropin levels (11). Thus, he fulfilled the criteria of CHH (1). The ~35 kb region shared by our proband and Xu’s patient (11) contained only a ribosomal pseudogene *RNA5SP293* (Fig. 2). This region also contains binding sites for ZNF143 (a transcription factor which interacts with a puberty-related factor, LIN28B, in neuroblastoma cells (50)), JUND (JunD binds to *Gnrhr* AP-1 site in mouse gonadotrope-derived αT3-1 cells (51) and is expressed in the mouse olfactory bulb (52)), GATA2 and GATA3 (crucial for neurogenesis and expressed in the olfactory bulbs (53)) and CEBPB (*Cebpb* in rats is expressed in the olfactory bulbs and olfactory ensheathing cells (54) and is known to be an upstream transcriptional regulator of *Gnrh1* in mice (55)). However, the mechanism by which a loss of transcription binding site(s) alone would cause disease is difficult to decipher, especially as the closest KS candidate gene locus (*PALM2AKAP2*) is relatively far away, 1.8 Mb, downstream from the 3’ breakpoint of the deletion. This locus in our patient with KS was, however, spared and expressed *PALM2AKAP2* efficiently (Fig. 3). Moreover, screening a set of Finnish KS patients revealed no pathogenic variants in *PALM2AKAP2*. Of note, the cases displayed in Fig. 2 showed wide phenotypic variability and we were able to verify KS-related features in only a proportion of them (Supplementary Table 2 and Supplementary Material).

Taken together, variation in the chromosomal region 9q31.2 at the population level associates with the variation in puberty timing and the sense of smell. Previous rare patients with deletions including 9q31.2 have exhibited CHH and KS-related phenotypes such as delayed puberty, an olfactory bulb defect, and cleft lip and/or palate. We describe the first patient with an extremely rare *de novo* deletion in 9q31.2 and KS. *ZNF462* haploinsufficiency was consistent with the patient’s Weiss–Kruszka syndrome but did not explain his KS. In further analyses, he did not carry rare sequence variants in known KS genes. Our results suggest that patients carrying a microdeletion in 9q31.2 should be evaluated for the presence of KS and KS-related features, and conversely, that KS patients with features of Weiss–Kruszka syndrome should be evaluated for the presence of a heterozygous 9q31.2 microdeletion.

## Supplementary Material

Supplementary Table 1. Two rare variants of uncertain significance in the whole exome sequencing data of the proband with a de novo microdeletion in 9q31.2 and Kallmann syndrome. Heterozygous variants in SARS1 and RIMBP3C were both inherited from the mother. Predicted protein change, variant type, and allele frequencies are shown.

Supplementary Table 2. Main clinical and genetic features of the patients listed in Figure 2.

Supplementary File Rare variants of uncertain significance detected in WES

## Declaration of interest

The authors declare that there is no conflict of interest that could be perceived as prejudicing the impartiality of this study.

## Funding

This work was supported by the Academy of Finland http://dx.doi.org/10.13039/501100002341, Foundation for Pediatric Research, Sigrid-Juselius Foundation, Novo Nordisk http://dx.doi.org/10.13039/501100004191 Foundation, Emil Aaltonen Foundation, University of Helsinki, Helsinki University Hospital, and Päivikki and Sakari Sohlberg foundation. Taneli Raivio is on the Editorial Board of European Journal of Endocrinology.

## Ethics committee approval

Informed consents were obtained from all patients, and in the case of a minor/children also a parent or guardian gave the consent. The study was approved by the Ethics Committee of the Hospital District of Helsinki and Uusimaa and was conducted in accordance with the Declaration of Helsinki.

## Author contribution statement

T R, V S, K V collected the study subjects and the clinical data and phenotyped the patients. J K, A-I P, V Y, H A, K V and T R analyzed the next-generation sequencing data, H A carried out ANNOVAR annotation, A-I P and K V performed Sanger sequencing data analyses. V Y performed *PALM2AKAP2* RT-qPCR analyses; A-I P, J K, V Y, K V and T R wrote the manuscript, and all authors critically revised the manuscript. T R coordinated and managed the study.
