# Supplementary material for: Kallmann syndrome in a patient with Weiss–Kruszka syndrome and a de novo deletion in 9q31.2
Source: Eur J Endocrinol. 2021 Apr 28;185(1):57–66. doi: 10.1530/EJE-20-1387 (PMC8183635; doi:10.1530/EJE-20-1387)
Supplement: Supplementary Table 1. Two rare variants of uncertain significance in the whole exome sequencing data of the proband with a de novo microdeletion in 9q31.2 and Kallmann syndrome. Heterozygous variants in SARS1 and RIMBP3C were both inherited from the mother. Predicted protein change, variant type, a [file supplementary_table_1.pdf]

Supplementary Table 1. Two rare variants of uncertain significance in the whole exome sequencing data of the proband with a *de novo* microdeletion in 9q31.2 and Kallmann syndrome. Heterozygous variants in *SARS1* and *RIMBP3C* were both inherited from the mother. Predicted protein change, variant type, and allele frequencies are shown.

Gene	Nucleotide change	Protein change	Frequency*
RIMBP3C	NC_000022.10:g.21904075_21904077del	p.(Glu397del)	-
SARS1	NC_000001.10:g.109778034G>A	p.(Arg317Gln)	0.000008[1]

*In ExaC, gnomAD, 1000 Genomes, dbSNP[1], or Exome Variant Server
