# Supplementary material for: Kallmann syndrome in a patient with Weiss–Kruszka syndrome and a de novo deletion in 9q31.2
Source: Eur J Endocrinol. 2021 Apr 28;185(1):57–66. doi: 10.1530/EJE-20-1387 (PMC8183635; doi:10.1530/EJE-20-1387)
Supplement: Supplementary Table 2. Main clinical and genetic features of the patients listed in Figure 2. [file supplementary_table_2.pdf]

Supplementary Table 2. Main clinical and genetic features of the patients listed in Figure 2.

**Publication, patient (reference)**	**Sex**	**Oldest reported age at clinical examination**	**Puberty details**	**Inheritance of the heterozygous deletion**	**Reported main phenotypic features**	**Reported genetic methodology***
Xu et al. 2013 (ref. 11)	M	20 years	started at 18 years, G2 at the age of 20 years	*de novo*	**Kallmann syndrome-related**: congenital hypogonadotropic hypogonadism<br>**other**: KBG syndrome (developmental delay, growth retardation, craniofacial phenotype, skeletal anomalies, hearing loss, dental anomalies) due to a mutation in *ANKRD11***	Sanger sequencing of *ANKRD11,* CGH array, chromosome analysis (550 G-band karyotyping)
Ramineni et al. 2019 (ref. 15)	M	19 years	delayed puberty at 15 years	from the affected mother	**Kallmann syndrome-related**: delayed puberty, hearing loss<br>**other**: delayed motor development, muscle pain, and cramps, short stature	chromosome microarray
Dugan et al. 2018, patient 2 (ref. 14)	M	10 months	prepubertal age	not mentioned	**Kallmann syndrome-related**: bilateral cleft lip and palate, renal hypoplasia, hypoplastic olfactory bulbs, and sulci<br>**other**: developmental delay, hypotonia, craniofacial anomalies, ptosis, ventriculomegaly, hearing impairment, mildly tapering fingers, and toes	SNP microarray
Cao et al. 2015 (ref. 13)	F	18 months	prepubertal age	*de novo*	**Kallmann syndrome-related:** unilateral cleft lip<br>**other**: Cornelia de Lange syndrome, short stature, developmental delay, craniofacial anomalies, hearing disorder, irregular dentition**, short neck**	WES, 550 G-band karyotyping, CGH Array, CNV validation with qPCR

Chien et al. 2010 (ref. 10)	F	6 years	prepubertal age	from the unaffected mother***	**Kallmann syndrome-related:** unilateral cleft lip and palate<br>**other**: mental retardation, craniofacial anomalies, strabismus, tapered fingers, low-set ears, mitral valve prolapse, delayed motor development, hearing impairment, postnatal growth restriction	G-band karyotyping, FISH, SNP array
Dugan et al. 2018, patient 1 (ref. 14)	F	11 years****	not mentioned	*de novo*	**Kallmann syndrome-related:** not reported<br>**other**: developmental delay, short stature, growth hormone deficiency, pituitary lesion, type 1 diabetes mellitus	SNP microarray
Mucciolo et al. 2014, patient 1 (ref. 12)	F	15 years 4 months	not mentioned	*de novo*	**Kallmann syndrome-related**: not reported<br>**other**: obesity, developmental delay, craniofacial anomalies, small hands with tapering fingers, a bicuspid aortic valve with moderate aortic insufficiency, short neck, cervico-thoracic gibbus	CGH array, microsatellite analysis of polymorphic loci in the deletion, sequencing of *FKTN,* cloning of breakpoints
Mucciolo et al. 2014, patient 2 (ref. 12)	F	29 years	not mentioned	*de novo*	**Kallmann syndrome-related:** not reported<br>**other**: obesity, mild intellectual disability, craniofacial anomalies, a small right clavicle, hepatosteatosis, hypercholesterolemia, type 2 diabetes mellitus, hypertension, dilated cardiomyopathy**,** cervico-thoracic gibbus, short neck**,** short stature	CGH array, microsatellite analysis of polymorphic loci in the deletion, sequencing of FKTN, cloning of breakpoints
Mucciolo et al. 2014, patient 3 (ref. 12)	F	not mentioned	not mentioned	*de novo*	**Kallmann syndrome-related**: not reported<br>**other**: mild intellectual disability, craniofacial anomalies, mild sensorineural hearing loss, hepatosteatosis, hypercholesterolemia, type 2 diabetes mellitus, hypertension, mild left ventricle hypokinesia**,** cervico-thoracic gibbus, short stature	CGH array, microsatellite analysis of polymorphic loci in the deletion, sequencing of *FKTN*, cloning of breakpoints
Weiss et al. 2017, patient in pedigree 4 (ref. 3)	F	5 years	prepubertal age	*de novo*	**Kallmann syndrome-related:** not reported<br>**other**: hypotonia, craniofacial anomalies, strabismus, corpus callosum dysgenesis, ptosis	chromosomal microarray

Weiss et al. 2017, patient in pedigree 5 (ref. 3)	F	12 years****	not mentione d	*de novo*	**Kallmann syndrome-related:** not reported **other**: ventricular septal defect, hyperopia, increased appetite, high pain threshold, mild intellectual disability, fine and gross motor delay, autism spectrum disorder, attention deficit disorder, obsessive-compulsive disorder, malformed and discolored teeth, overweight	chromosomal microarray
Kulharya et al. 2008, patient 2, (ref. 9)	F	4 months	prepubert al age	*de novo*	**Kallmann syndrome-related**: not reported **other**: developmental delay, craniofacial anomalies, protruding ears, narrow palate and a short neck, short stature	cytogenetic and FISH analysis, CGH array, SNP array

*Cao et al. applied whole exome sequencing (WES) but do not report variants in genes implicated in Kallmann syndrome. Other studies do not report Kallmann syndrome-related genetic findings

**_ANKRD11_ mutations predispose to precocious puberty (supplementary ref. 5)

***Patient's deletion (del(9)(q31.1q33.1)) was inherited from her unaffected mother with an inverted insertion (18;9)(q12.2;q33.1q31.1)

****Reported age within or at the borderline of the normal limit (± 2SD; 8 years - 12 years) for Tanner breast stage M2 (supplementary ref. 6)
