# Supplementary material for: Kallmann syndrome in a patient with Weiss–Kruszka syndrome and a de novo deletion in 9q31.2
Source: Eur J Endocrinol. 2021 Apr 28;185(1):57–66. doi: 10.1530/EJE-20-1387 (PMC8183635; doi:10.1530/EJE-20-1387)
Supplement: Supplementary File Rare variants of uncertain significance detected in WES [file supplementary_material.pdf]

Supplementary File

Rare variants of uncertain significance detected in WES

The proband harbored two maternally inherited, rare heterozygous variants of uncertain significance detected in WES (Supplementary Table 1). The first variant lied in the RIMS-binding protein-3C gene (*RIMBP3C*), which is expressed mainly in the testis, interacts with the Hook1 cargo adaptor protein in mice, and is implicated in spermatogenesis (supplementary ref. 1). However, expression *of RIMBP3C* is very low in the human central nervous system and pituitary gland (Human Protein Atlas), and *Rimbp3* knock-out male mice exhibit similar weights of seminal vesicles, testes, and cauda epididymis to WT mice, indicating normal genital development (supplementary ref. 1). The second variant was in *SARS1* (encoding seryl-tRNA synthetase-1). Biallelic pathogenic variants in *SARS1* were reported in an Iranian family with syndromic intellectual disability, microcephaly, ataxia, and seizures (supplementary ref. 2). Moreover, a *de novo* heterozygous variant in *SARS1* has been reported in a patient with sporadic non-syndromic brain arteriovenous malformation (supplementary ref. 3). According to the 2015 ACMG/AMP guidelines (supplementary ref. 4), the *SARS1* and *RIMBP3C* variants were classified as variants of uncertain significance, as the associations of the *SARS1* and *RIMBP3C* genes to the proband's phenotype are uncertain.

Patients in Figure 2

Features associated with KS were reported in five patients (Xu et al., 2013; Ramineni et al., 2019; Dugan et al., 2018 (patient 2); Cao et al., 2015; and Chien et al., 2010). At the time of assessment, five patients (Xu et al., 2013; Ramineni et al., 2019; and all three patients in Mucciolo et al., 2014), had reached pubertal age, and delayed puberty was diagnosed in the patient in Ramineni et al., 2019 and CHH in the patient in Xu et al., 2013.

All patients, except for the family members reported by Ramineni et al., 2019, and Dugan et al., 2018 (patient 1), presented with additional various craniofacial phenotypes. Moreover, the inheritance of the deletion was reported in all but in patient 2 in Dugan et al., 2018. Known CHH/KS genes were sequenced only in one patient (Cao et al., 2015), and no mutations in CHH/KS genes were reported (Supplementary Table 2).

Supplementary references

1. Zhou J, Du YR, Qin WH, Hu YG, Huang YN, Bao L, Han D, Mansouri A, Xu GL. RIM-BP3 is a manchette-associated protein essential for spermiogenesis. *Development* 2009 **136** 373-382

2. Musante L, Püttmann L, Kahrizi K, Garshasbi M, Hu H, Stehr H, Lipkowitz B, Otto S, Jensen LR, Tzschach A et al. Mutations of the aminoacyl-tRNA-synthetases SARS and WARS2 are implicated in the etiology of autosomal recessive intellectual disability. *Hum Mutat* 2017 **38** 621-636.

3. Wang K, Zhao S, Liu B, Zhang Q, Li Y, Liu J, Shen Y, Ding X, Lin J, Wu Y et al. Perturbations of BMP/TGF-β and VEGF/VEGFR signalling pathways in non-syndromic sporadic brain arteriovenous malformations (BAVM). *J Med Genet* 2018 **55** 675-684.

4. Richards S, Aziz N, Bale S, Bick D, Das S, Gastier-Foster J, Grody WW, Hegde M, Lyon E, Spector E, Voelkerding K, Rehm HL; ACMG Laboratory Quality Assurance Committee. Standards and guidelines for the interpretation of sequence variants: a joint consensus recommendation of the American College of Medical Genetics and Genomics and the Association for Molecular Pathology. *Genet Med* 2015 **17** 405-424.

5. Goldenberg A, Riccardi F, Tessier A, Pfundt R, Busa T, Cacciagli P, Capri Y, Coutton C, Delahaye-Duriez A, Frebourg T, Gatinois V, Guerrot AM, Genevieve D, Lecoquierre F, Jacquette A, Khau Van Kien P, Leheup B, Marlin S, Verloes A, Michaud V, Nadeau G, Mignot C, Parent P, Rossi M, Toutain A, Schaefer E, Thauvin-Robinet C, Van Maldergem

L, Thevenon J, Satre V, Perrin L, Vincent-Delorme C, Sorlin A, Missirian C, Villard L, Mancini J, Saugier-Veber P, Philip N. Clinical and molecular findings in 39 patients with KBG syndrome caused by deletion or mutation of ANKRD11. *Am J Med Genet A* 2016 **170** 2847-2859.

6. Lindhardt Johansen M, Hagen CP, Mieritz MG, Wolthers OD, Heuck C, Petersen JH, Juul A. Pubertal progression and reproductive hormones in healthy girls with transient thelarche. *J Clin Endocrinol Metab* 2017 **102** 1001-1008.
